# Current State of Food Prescriptions Used to Treat Cardiometabolic Risk Factors in the US Adult Population

**DOI:** 10.7759/cureus.53629

**Published:** 2024-02-05

**Authors:** Karim Bourenane, Nora Emon

**Affiliations:** 1 Medicine, California University of Science and Medicine (CUSM) School of Medicine, Colton, USA; 2 Family Medicine, Kaiser Permanente Oakland Medical Center, Oakland, USA

**Keywords:** body mass index, hemoglobin a1c (hba1c), blood pressure, fruits and vegetable, food pharmacy, food prescriptions, cardiometabolic

## Abstract

Cardiometabolic syndrome is unfortunately widely prevalent in medically underserved areas with one possible non-pharmacological solution being food prescriptions from food pharmacies. Food prescriptions are defined as when a physician prescribes certain foods as a treatment for health conditions. There seems to be a promising future for food prescriptions; however, there is a huge literature gap. Given this lack of knowledge regarding this burgeoning practice, we decided to review the current state of food prescriptions used to treat cardiometabolic conditions in the US adult clinical setting. A thorough search of PubMed and Google Scholar databases for articles written about food prescriptions' impact on cardiometabolic risk factors was done. The keywords used included “food prescriptions, vegetables prescription, produce prescription, fruit prescriptions, food pharmacy, food as medicine, cardiometabolic, blood pressure, glucose, insulin, cholesterol, obesity, BMI, body mass index, triglycerides, and microalbuminuria." Of the 637 articles found with the associated keywords, 115 were kept after being screened by title and abstract. Finally, after a full-text record screening, 30 articles were deemed eligible based on our inclusion criteria. We analyzed the health markers, patient populations, methods of food procurement, and financial incentives in food prescription programs. On average, the implementation of food prescription programs decreased participants' BMI, waist circumference, blood pressure, and HbA1c. Participants in the programs were primarily comprised of African American, Hispanic, underinsured, low-income, older, and women groups. Programs with subsidies and vouchers had a higher compliance rate, and food sourced from farmers' markets, grocers, and mobile vendors had the best program compliance rates. According to the literature, adherence to food prescription programs on average decreases the BMI, blood pressure, waist circumference, and Hb1Ac of participants. However, those are the only biomarkers being studied currently, and future studies should incorporate other markers of chronic conditions. For example, a reliable indicator of cardiometabolic health is total cholesterol/HDL cholesterol, which should be measured in future experiments. Additionally, insulin, glucose, triglycerides, and LDL cholesterol are all great markers of cardiometabolic health that can be measured in the future. The current implementation of many food prescription programs is in medically underserved areas. The patient populations are typically low-income, under- or uninsured, food insecure, and originating from diverse ethnic backgrounds. In the future, food prescription studies should be done on other ethnic populations including but not limited to Native Americans who also carry a large burden of preventable and chronic illnesses.

## Introduction and background

It is no secret that a steady diet of vegetables and leafy greens creates a protective health effect in individuals with cardiometabolic risk factors and disease [1]. Despite the consensus regarding the many health benefits of integrating vegetables into one’s daily diet, most American adults still fail to eat the recommended amount. According to the CDC, only 12% of Americans meet the daily fruit requirement and only 9% meet the daily vegetable requirement [2]. In underserved communities, the failure to eat a healthy diet is even more pronounced due to a variety of factors including but not limited to poor health literacy, food deserts, and rising costs of fresh foods. Residents of poorer neighborhoods typically consume high-sugar, high-fat processed foods instead of healthier, albeit more expensive, options [3].

All the risk factors (lack of healthy food, poorer neighborhoods, medically underserved areas) serve as multipliers for the risk of cardiometabolic conditions [3]. Cardiometabolic syndrome is defined as a combination of metabolic dysfunctions mainly characterized by insulin resistance, impaired glucose tolerance, dyslipidemia, hypertension, and central adiposity [4].

Although many factors are involved in cardiometabolic disease, having a healthy diet is a modifiable risk factor associated with improved cardiometabolic health [5]. In fact, following healthy eating patterns is the gold standard for reducing mortality and increasing lifespan in cardiometabolic disease [6]. Diets high in fish, whole grains, fruits, vegetables, and legumes are considered to have cardioprotective properties, while diets high in refined sugar, trans-fats, sugar, and excessive salt are associated with poor cardiometabolic health [7]. The modifiable nature of the diet makes food consumption changes a popular goal for healthcare providers along with weight loss, smoking cessation, and lowering blood pressure [5].

One way to address cardiometabolic syndrome in medically underserved areas is food prescriptions from food pharmacies. Diet or food charts are general guidelines for what food groups an individual should and should not consume [8]. For example, the classic food pyramid and food plate provide general guidelines for what an individual should and should not consume from food groups like cereals, vegetables, meat, and so on [9]. Food prescriptions are defined as a physician’s recommendation to eat certain foods (by quantity and frequency) as a treatment for health conditions. These healthy foods can be obtained by a voucher, for free, or out of pocket. In medically underserved areas, food prescriptions have been increasing in popularity as non-pharmacological interventions to improve the risk factors for chronic illnesses. Notably, food prescriptions are not used alone and are often part of a comprehensive treatment plan that also includes medications, lifestyle changes, and regular medical monitoring.

With the end of the Emergency Supplemental Nutritional Assistance Program and the rising costs of groceries brought about by inflation, food prescriptions are becoming an increasingly attractive treatment option. A study using a simulation model estimated that just a 30% subsidy food and vegetable incentive would prevent 1.93 million cardiovascular events, prevent 0.35 million cardiovascular deaths, and save 40 billion dollars in healthcare costs [10]. This was regardless of age, ethnicity, education, income, and participation in the Supplemental Nutritional Assistance Program. There seems to be a promising future for food prescriptions; however, a huge gap in the scientific literature regarding them remains. 

A meta-analysis that studied the context, analysis, and scope of food prescription programs found that there are questions about food programs’ long-term effectiveness, impact on health behaviors, screening of eligible participants, and logistics of program implementation [11]. Additionally, a systematic review of articles regarding food prescriptions' impact found that most studies conducted on food programs’ effectiveness had small sample sizes [12]. Additionally, medical providers themselves would like to use food prescriptions, but there is a lack of understanding of how food prescriptions would be implemented and their health outcomes [13]. Considering this lack of knowledge regarding this burgeoning practice, we decided to review the current state of food prescriptions used to treat cardiometabolic conditions in adults in the US clinical setting. There is no sole definition for cardiometabolic risk factors; however, we decided to use the most widely used criteria defined by the World Health Organization and National Cholesterol Education Program. This definition includes an increased BMI/waist circumference, insulin-resistant glucose metabolism, and decreased HDL cholesterol [4].

## Review

Methods

Inclusion Criteria

A thorough search of PubMed and Google Scholar databases for articles written about food prescriptions’ impact on cardiometabolic risk factors was conducted. The PubMed database was included because of the widespread use of systematic reviews and Google Scholar as it is readily accessible owing to its increasing coverage of scientific literature [14]. A systemic review studying the effect food prescriptions had on chronic illness management in low-income populations also used PubMed and Google Scholar for their meta-analysis [12]. Keywords used included “food prescriptions, vegetables prescription, produce prescription, fruit prescriptions, food pharmacy, food as medicine, cardiometabolic, blood pressure, glucose, insulin, cholesterol, obesity, BMI, body mass index, triglycerides, and microalbuminuria.” Of the 637 articles found with associated keywords, 115 were kept after being screened by title and abstract. Finally, after a full-text record screening, 30 articles were deemed eligible based on our inclusion criteria.

Exclusion Criteria

**Figure 1:**
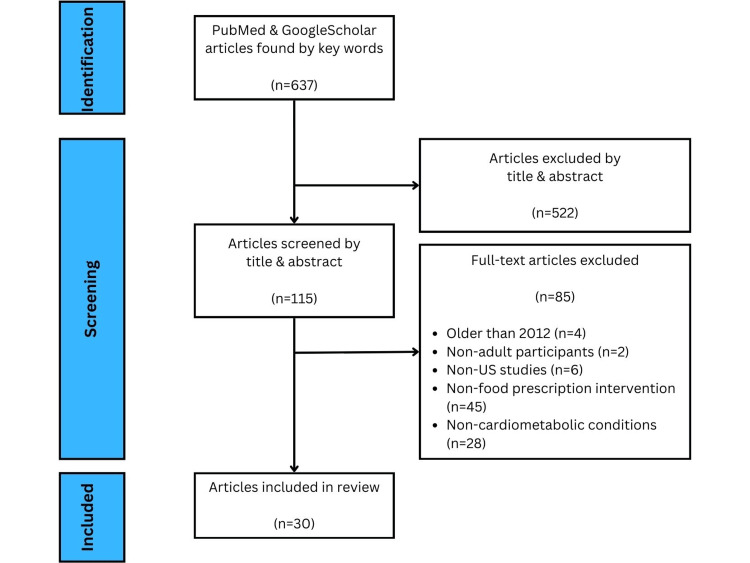
PRISMA flow diagram for article selection PRISMA: Preferred Reporting Items for Systematic Reviews and Meta-Analyses

Articles or studies are needed to analyze the US adult population. Studies done internationally or on pediatric populations were excluded. Articles are needed to study the impact of food prescriptions on cardiometabolic risk factors. Articles studying the impact of food prescriptions on non-cardiometabolic-related risk factors and illnesses were excluded. Articles older than those published in 2012 were excluded to cover only up-to-date studies. Articles with non-food prescription interventions were excluded. Figure 1 illustrates the process of article selection and exclusion.

Results

We analyzed the patient populations, experimental design, food procurement, participation incentives, and results of each study relevant to our meta-analysis. 

A large-scale pre- and post-survey conducted across several states recruited 2064 adult participants, with 70.7% being women and the average age being 54.4 years. Adults with obesity, prediabetes, and/or diabetes were recruited. Children were also recruited in this large-scale study, but they will be excluded from our analysis as per our inclusion criteria. Thirty percent of the adults were non-Hispanic White, 45% were Black, and 21% were Hispanic. The adult food prescription programs were conducted in Florida, Ohio, Connecticut, North Carolina, Georgia, Minnesota, Colorado, California, Texas, and New York. Participants received a variety of incentives ranging from $15 to $300 per month to purchase fresh fruits and vegetables from various sites. Participants with poor cardiometabolic health were defined as adults with a BMI greater than or equal to 25, systolic blood pressure greater than or equal to 130, diastolic blood pressure greater than or equal to 80, and an HbA1c greater than or equal to 6.5%. Participants with poor cardiometabolic health saw their systolic blood pressure drop by 8.38 mmHg and diastolic pressure drop by 4.94 mmHg. Their HbA1c decreased by 0.29, and their BMI fell to 0.36 kg/km^2^. The effect was even more pronounced in participants with very poor cardiometabolic health, which was defined as adults with a BMI greater or equal to 30, systolic blood pressure greater than or equal to 140, diastolic blood pressure greater or equal to 90, and a HbA1c greater than or equal to 8.0%. This group saw their systolic and diastolic pressure drop by 11.10 mmHg and 9.43 mmHg, respectively. Their HbA1c decreased by 0.58 and their BMI fell 0.52 kg/km^2^ [15].

A pre- and post-survey studied the impact of a 13-week Fresh Prescription Program at a federally qualified health center in Southeastern Michigan on 65 low-income patients with uncontrolled diabetes. All participants were low-income and had uncontrolled diabetes indicated by their high HbA1c levels. Sixty-six percent of the subjects were Hispanic/Latino, 28% were Black/African American, and the remaining participants were White. Most subjects were women, with a mean age of 52.5 years. Forty percent of the patients were uninsured, 55.4% were covered by Medicaid or Medicare, and 4.6% were covered by commercial insurance. The participants had a significant decrease in BMI, blood pressure, and Hb1Ac. The magnitude of improvement of Hb1Ac was even comparable to a person on glucose-lowering medications [16].

An interventional study recruited 49 patients from Hayward, California, with poor social determinants of health and conditions like cardiovascular disease, diabetes, and depression. The patients were mostly women with an average age of 59.1 years who came from ethnically diverse backgrounds (African American, Hispanic/Latino, Pacific Islander, and Caucasian). The patients were given access to a food pharmacy on top of nutritional coaching and exercise. Ten-dollar vouchers for farm stands in the clinic’s lobby were provided. All 49 participants were found to be food insecure. They suffered from a variety of conditions including diabetes, prediabetes, cardiac disease, hypertension, dyslipidemia, obesity, and anxiety. The study was carried out at a Federally Qualified Healthcare Center, which covers mostly Medicaid patients and the uninsured. There was a significant decrease in BMI, systolic blood pressure, and depressive symptoms. Participants were found to have increased daily servings of vegetables and fruits. Although participants were given additional interventions alongside healthy food, this still demonstrates the positive impact of regular healthy eating for improving chronic conditions [17]. 

A retrospective case-control study design using medical records evaluated a food prescription program in a low-income urban neighborhood in Albany, New York. Approximately 53.7% of the 54 participants were African American, 29.6% were Caucasian, 3.7% were biracial, and the remaining 13% did not have a race or ethnicity documented. Eighty-eight percent of the subjects were covered by Medicare and or Medicaid, with the remaining 12% either having private insurance or no insurance at all. Patients were given a total of 13 coupons with each coupon having $7 of value for a total of $91, which could be used to buy produce from a mobile produce market known as “Capital Roots Veggie Mobile” that travels throughout inner-city neighborhoods. The participants had a small but notable decrease in BMI by 0.74 kg/m^2^ [18]. 

A quasi-experimental design conducted in the Harris Health Strawberry Center, a primary care clinic, in Harris County, Texas, recruited 42 participants who were food insecure and had an HbA1c level greater than 7%. Participants were given 30 pounds of free fruits and vegetables on a biweekly basis from the Harris Health Strawberry Center food pantry. Notably, participants were also given access to virtual nutrition education while they were receiving free produce. Eighty percent of the participants were women with the average age being 57 years. Approximately 86.2% of the participants were Latino American, Hispanic, or of Spanish origin. There was an average reduction of HbA1c of 0.96 among participants in the program. Although data were collected regarding the change in systolic and diastolic blood pressure and BMI, there were no significant reductions observed [19]. 

A pre- and post-survey conducted in Santa Barbara County, California, recruited 159 participants via bilingual outreach, with 75% of them being Hispanic/Latino with high rates of diabetes and prediabetes. Approximately 76.7% of participants were women, and the mean age was 52.5 years. Additionally, 35% of the participants were uninsured. Participants collected free weekly produce prescriptions for 10 weeks from farms sourced within a 72-mile radius. Waist circumference was found to significantly decrease by 1 cm, and systolic blood pressure significantly dropped by 2.4 mmHg. There was no significant change in the participants’ BMI, diastolic blood pressure, weight, and HbA1c; however, there was less blood glucose variability [20]. 

A one-year randomized control trial recruited 298 participants with hypertension and/or hyperlipidemia from a southeastern part of North Carolina. Eighty-four percent of the participants were women, and the average age was 72.4 years. Two-thirds of the population were white, and an overwhelming majority (91%) of the patients were not on Medicaid. Participants received seven, frozen Dietary Approaches to Stop Hypertension (DASH)-compliant diets per week for one year. The DASH diet is a low-salt diet used to decrease high blood pressure and LDL cholesterol [21]. There were no significant changes in biomarkers (BMI and energy intake) found across groups attributable to the food prescription program even going by subgroup [22].

Another retrospective case-control study recruited 423 participants from the Greater Houston Area via the Houston Food Bank. The average age was 57 years, and about 70% of the participants were women. Fifty-six percent of the participants were Hispanic, 32% were African American, and nearly 80% reported being food insecure. Participants were provided with food prescriptions, which allowed up to 30 pounds of fresh produce to be redeemed on a bimonthly basis. Participants were found to have a significant decrease in systolic blood pressure by 3.2 mmHg and a decrease in diastolic blood pressure by 2.5 mmHg. HbA1c levels declined by 0.52 in participants who redeemed their prescriptions at least once a month. There were no changes in BMI or LDL levels post-study [23]. A summary of our key findings can be found in Table 1.

**Table 1:** Studies focusing on food pharmacies’ impact on cardiometabolic markers BMI: body mass index; SBP: systolic blood pressure; DBP: diastolic blood pressure; HbA1c: glycated hemoglobin; ↓: decreased

Study	Design	Results
Hager et al. [15]	Cohort	↓BMI 0.52 kg/m^2^, ↓SBP 11.10 mmHg, ↓DBP 9.43 mmHg, ↓HbA1c 0.58
Bryce et al. [16]	Cohort	↓HbA1c 0.71, No significant change in weight or SBP or DBP
Emmert-Aronson et al. [17]	Cohort	↓BMI 0.10 kg/m^2^, ↓SBP 1.68 mmHg, No significant change in DBP
Cavanagh et al. [18]	Case-control	↓BMI 0.74 kg/m^2^
Sharma et al. [19]	Cohort	↓HbA1c 0.96, No significant change in SBP or DBP or BMI
Kerr et al. [20]	Cohort	↓Waist circumference 1 cm, ↓SBP 2.4 mmHg, No significant change in DBP or BMI
Racine et al. [22]	Randomized controlled trial	No significant change in BMI
Ranjit et al. [23]	Case-control	↓SBP 3.2 mmHg, ↓DBP 2.5 mmHg, ↓HbA1c 0.52

Discussion

Research into the efficacy of food prescription programs is a relatively new venture that leaves much to be desired in terms of conducting large randomized controlled trials to establish cause and effect between food prescriptions and improved health outcomes. However, food pharmacies almost always increase the vegetable and food consumption of their participants. Bhat et al. [24] investigated 13 randomized controlled trials in a meta-analysis that found that participants categorically ate more vegetables and fruits.

Studies done regarding food prescriptions typically measure several biomarkers of health such as blood pressure, BMI, HbA1c, and weight as ways to objectively measure the impact of these food programs on patients. Adherence to healthy food programs on average seems to decrease the values of these biomarkers, which are associated with positive health outcomes. A meta-analysis reviewing interventional studies in 2021 that looked into the effect food prescriptions had on health outcomes found that BMI, HbA1c, and blood pressure decreased significantly in participants who increased their fruit and vegetable intake through food prescription programs [24]. Another key finding was that the improvements in dietary behaviors and cardiometabolic risk factors were most pronounced in individuals with obesity and low socioeconomic status.

According to the literature, adherence to food prescription programs decreases the BMI, blood pressure, Hb1Ac, and weight of participants. This excludes the study by Racine et al. [22], which did not observe significant changes, and the researchers believe it may have been because of the sample size coming only from the southeastern urban area of the US and not being representative of the adult population. Notably, this study was a randomized control trial done in 2012, and more current randomized control trials need to be conducted on food prescription programs. However, BMI, blood pressure, HbA1c, and weight seem to be the main biomarkers being studied currently, and future studies should incorporate other markers of chronic conditions. For example, a reliable indicator of cardiometabolic health is total cholesterol/HDL cholesterol, which should be measured in future experiments [25]. Additionally, insulin, glucose, triglycerides, and LDL cholesterol are all great markers of cardiometabolic health that can be measured in the future [26].

Most of the studies regarding the impact of food prescriptions are conducted on patients coming from medically underserved areas. This makes sense as these areas lack adequate healthcare access and stand the most to gain from food prescriptions. Patient populations are typically low-income, non-White, under or uninsured, food insecure, and suffer from chronic illnesses. We also noted that most of the participants in these studies are women over 50 years old, and further studies may elucidate the reason for this specific demographic being consistently the largest group in food prescription studies.

Several barriers prevent food prescription programs from being completely effective including but not limited to cost, transportation, health literacy, geographic access, and social support [27]. Affordability of fruits and vegetables is a very common barrier for working-class Americans, and so many food pharmacy programs provide vouchers, gift cards, or cash for participants to redeem. Mobile food vendors have been created in an attempt to go to underprivileged areas with a lack of transportation. Many food pharmacy programs have included health literacy courses with their food prescriptions to increase health and food literacy. Food prescription utilization may increase if barriers of stigma, transportation, and poor nutrition literacy are addressed [28].

Studies conducted on the efficacy of food pharmacies have yet to describe in detail the complex demographics of their adult participants, which we laid out above for each relevant study. The meta-analysis of PubMed and Google Scholar food pharmacy studies discussed earlier reported on the ethnic background, age, and sometimes Medicaid and Supplemental Nutrition Assistance Program enrollment of participants of the studies included in their review article [12]. A systematic review done in 2021 regarding the strengths and limitations of food pharmacy studies described the financial status of their adult and child participants, but it did not go into detail regarding other demographic information [29]. Future studies should continue to further describe the demographics of participants including but not limited to race, ethnicity, gender, sex, income, insurance status, employment status, and age. To our knowledge, no studies have been conducted on Native American adults, except a study done by Jones et al. on Navajo children that found that the program increased the consumption of fruits and vegetables among the participants [29]. This will give researchers a better idea of who is using food pharmacies and where their placement can be the most effective. If large-scale studies consistently find significant impacts on the health outcomes of study participants, then food prescriptions should be seriously considered to be integrated into the healthcare system as a viable auxiliary to medical management for cardiometabolic conditions [30].

Ideally, future studies will have a random controlled trial design rather than retrospective studies to establish greater certainty in the causality of food prescription programs. We acknowledge that these experimental designs are much more rigorous and harder to conduct. If possible, larger sample sizes should be collected to allow for more external validity. Hager et al. [15] could serve as a future template for analyzing food prescription studies across multiple states as they collected information from 10 states. 

A systematic review that analyzed 14 food prescription studies conducted among adults and children found the unique characteristics of the most effective programs. Programs that ranged between three and six months encouraged multiple patient visits and provided financial incentives saw the most participation and success. Additionally, effective studies sourced their food from mobile vendors, farmer’s markets, and local grocers. A large limitation noted by this review was the lack of follow-up after the studies were conducted [26]. Based on this analysis, longitudinal studies should be conducted to see if participants continue with their dietary changes, and if large changes are seen in biomarkers such as HbA1c, blood pressure, BMI, etc. in the long term. 

## Conclusions

With the adult American population suffering from many chronic and preventable illnesses, food prescription programs may be a promising auxiliary to the traditional management of cardiometabolic conditions. Food subsidies have been shown to prevent cardiac events and deaths and save the healthcare system billions of dollars. With this promising future, the literature regarding food pharmacy efficacy remains in its infancy, and more up-to-date randomized control trials with large sample sizes must be conducted to see if these programs should be implemented at scale. Food prescriptions so far have been shown on average to decrease participants’ BMI, blood pressure, weight, and HbA1c. Future studies should investigate more markers of health in cardiometabolic conditions such as LDL, HDL, triglycerides, and so on.
